# Impacts of some metal ions on glutathione s-transferase in the liver of *Chalcalburnus tarichi*: an endemic species of Lake Van

**DOI:** 10.55730/1300-0527.3439

**Published:** 2022-05-11

**Authors:** Muhammet Serhat ÖZASLAN, Mehmet ÇİFTCİ

**Affiliations:** 1 Department of Pharmacy Services, Nihat Delibalta Göle Vocational High School, Ardahan University, Ardahan, Turkey; 2 Department of Chemistry, Faculty of Sciences, Bingöl University, Bingöl, Turkey

**Keywords:** Lake Van fish, purification, glutathione S-transferase, inhibition

## Abstract

Glutathione-S-transferase (GSTs) is a multifunctional enzyme that provides homeostasis by catalyzing the first step of the formation of mercapturic acid, the end product in detoxification metabolism. They can prevent reactive electrophilic compounds from harming the body by covalently binding the same type of compounds to each other. In this study, we determined the in vitro inhibitory effects of metal ions such as Cu^2+^, Cd^2+^, Ag^+^, and Co^2+^ on GST enzyme activity. For this aim, GST was purified from *C. tarichi* Pallas liver with 37.36% yield and 29.304 EU/mg specific activity using the chromatographic method. The V_max_ values of liver GST were determined for CDNB and GSH to be 1.245 and 0.562 EU/mL, respectively, and the K_m_ values were found 0.89 and 0.06 mM, respectively, using the Lineweaver–Burk plot. The effects of the metal ions at different concentrations on in vitro GST activity were studied. The IC_50_ values were determined for Cu^+2^, Cd^+2^, Ag^+^, and Co^+2^ as 0.163, 0.235, 0.00021, and 0.446 mM, respectively. The K_i_ constants were determined as 0.049 ± 0.009, 0.117 ± 0.031, 0.002 ± 0.0007, and 0.893 ± 0.3 mM, respectively. Ag^+^ showed the best inhibitory effect among the studied metal ions. Cd^2+^, Cu^2+^ and Co^2+^ showed a competitive inhibition mechanism, while Ag^+^ was noncompetitive.

## 1. Introduction

With industrialization, our world is facing many threats. One of the most important of these threats is water pollution [1]. Pollution in water is examined in three groups according to the type of pollutant as physical, chemical, and biological pollution. It was reported that water is rapidly polluted in an irreversible way due to pollutant sources such as mining enterprises, industry, industrial wastes, agricultural wastes, food industry wastes, sewage wastes, and natural disasters [2]. Some sources of chemical pollution are transition elements, which are called heavy metals with atomic numbers greater than 20 and have a toxic effect and a density of more than 5 g/cm^3^ [3]. Heavy metals pass into aquatic environments such as rivers and lakes with factory wastes, dust carried by the wind, forest fires, volcanic activities, rock fragments carried by erosion, domestic wastes, and sewers, and they harm organisms living in these environments in different ways [4]. Heavy metals entering aquatic environment such as seas, lakes, and rivers cause bioaccumulation in fish. Although some heavy metals are necessary at low concentrations for organisms to continue their vital activities, they can also pass through the food chain to those who feed on these organisms and affect their health negatively [5].

Fish take heavy metals in from their external environment through gills, food, and skin [6,7]. Heavy metals taken by fish from their external environment are carried to tissues and organs by blood by clinging to transporter proteins, and they can reach high rates by clinging to metal-retaining proteins in tissues [8]. These pollutants acquired from the external environment are mostly bound to proteins and transmitted via blood to storage sites or the liver for transformation or storage. Heavy metals transformed by the liver are stored here, sent to the bile duct, or returned to blood to be excreted by the kidneys [9].

Oxidative stress can cause a disturbance in the oxidant–antioxidant balance, leading to effective tissue and cellular damage [10,11]. During oxidative phosphorylation, reactive oxygen species and other so called ‘free radicals’ are constantly produced in vivo by all body tissues in the mitochondrial matrix [12,13]. Recently, biomarkers of oxidative stress have been a significant topic of studies in this field. As oxidative stress markers, there are several parameters that can be measured [14]. These markers may be enzymatic markers such as glutathione S-transferase (GST) and nonenzymatic antioxidants including 8-hydroxy-2-deoxyguanosine levels and total thiol levels [15].

Glutathione S-transferases (GSTs) are detoxification isoenzymes and have a significant role in cellular signaling [16,17]. Human GSTs (hGSTs) are classified into three types, membrane-associated proteins, the mitochondrial type and the cytosolic type based on their cellular localization [18]. The mitochondrial and cytosolic types are divided into eight classes as alpha, mu, pi, theta, omega, zeta, sigma, and kappa, and they possess some primary structural similarities [19].

This study focuses on the purification and characterization of *C. tarichi* Pallas liver GST activity and kinetics of in vitro GST inhibition by Cd^2+^, Cu^2+^, Co^2+^, and Ag^+^ metal ions.

## 2. Materials and methods

### 2.1. Sample preparation and enzyme purification

*C. tarichi* Pallas fish were obtained from Van Lake basin. The liver samples of *C. tarichi* Pallas were immediately removed and preserved for experimental studies according to the cold chain principles. The livers were sliced into small pieces and successfully lysed by a homogenizer in 50 mM Tris/HCl buffer (pH 7.2) containing 1 mM DTT, 1 mM PMSF and 1 mM EDTA. Homogenate was centrifuged twice at 4 °C and 20,000 × *g* for 60 min. The supernatant was used in subsequent analyses. For GST purification, we followed the procedure described in our previous study [9].

### 2.2. Enzyme assay

GST activity was measured with the method in our previous study [9] using CDNB as the model substrate.

### 2.3. Protein quantity assay and characterization of GST enzyme

Bradford procedure was used for protein quantity [20]. The optimal pH and ionic strength were determined based on the activity of the pure enzyme described in a previous study [21]. The effects of temperature on GST activity were made spectrophotometrically at temperatures ranging from 0 to 80 °C as described in a previous study [22]. Stable pH ranges were 5.5–8.0 and 7.0–9.0. The enzyme solution was mixed with the buffers at the corresponding pH values. The activities were assayed at every 24 h for 4 days [23]. Different concentrations of GSH and CDNB were used as substrates to determine the V_max_ and K_m_ values of the GST enzyme. V_max_ and K_m_ values were determined based on the Lineweaver–Burk plot [24].

### 2.4. SDS-polyacrylamide gel electrophoresis

The purity of GSTs was observed by the SDS-PAGE technique according to Laemmli’s procedure [25]. The method was performed according to information in previous studies [26–28].

### 2.5. In vitro inhibition effect of metals on GST enzyme activity

IC_50_ was defined as the concentration of the compound causing 50% inhibition, and it was calculated based on Activity (%)–[metal ion] plots for each metal [29–32]. The K_i_ values and inhibition types were found using Lineweaver–Burk plots [33–35]. For the K_i_ study, we selected 3 inhibitor concentrations and made activity measurements relative to the control values, and then we calculated 1 / S and 1 / V values and plotted K_i_ plots. Analysis of the data and drawing of graphs were realized using GraphPad Prism ver. 8 for Mac (GraphPad Software, La Jolla California USA). The results were exhibited as mean ± standard error of the mean (95% confidence intervals). Differences between data sets were considered statistically significant when the p-value was less than 0.05.

## 3. Results and discussion

GSTs play a key role in the metabolism and synthesis of endogenous and exogenous compounds that include an electrophilic carbon, sulfur, or nitrogen atom. Their substrates contain arene oxides, α,β-unsaturated carbonyls, halogenonitrobenzenes, and quinones. During oxidative stress, GSTs have also been situated to intercede signal transduction and reduce the impacts of oxygen toxicity [15–28]. To the best of our knowledge, the effects of metals on the GST enzyme activity of the livers of fish living in Lake Van have not been studied yet.

In this study, the GST enzyme was purified from *C. tarichi* Pallas liver with 37.36% yield and 29.304 EU/mg specific activity (Table 1).

**Figure 1:**
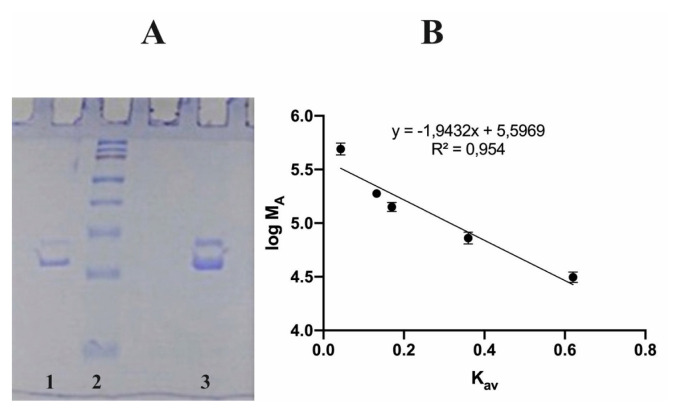
A-) SDS-PAGE analysis of purified GST. (1: Purified enzyme, 2: Startdard proteins 130 kDa, 100 kDa, 70 kDa, 55 kDa, 45 kDa, 35 kDa, 25 kDa, 15 kDa, 3: Purified enzyme) B-) Standard K_av_-log M_W_ graph of GST using Sephadex G-150 chromatography.

The purification procedure was performed in one step. This procedure is simple and useful, and it can be employed for other studies. According to SDS–PAGE, GST showed a heterodimeric structure, and the molecular weights of the subunits were determined as 28.00 and 33.80 kDa. The natural molecular weight of GST was found as 62.35 kDa using the Sephadex G-150 gel filtration chromatography method based on the K_av_-Log M_w_ plot (Figure 1).

In the literature, researchers have purified GST from various tissues of diverse fish species under laboratory conditions. For example, Donham et al. [36] purified GST from *P. californicus* liver with 0.23 EU/mg specific activity. In another study, Lee et al. [37] cloned and purified a theta-class-like glutathione S-transferase (GST-T) from the liver tissue of *R. marmoratus* with 9.94 ± 0.17 EU/mg specific activity and 29.48% yield. Our results determined that GST was purified with a high specific activity in comparison to studies of various fish tissues and species in the literature.

In our study, the characteristic properties of the GST enzyme were determined. The optimum temperature, ionic strength, pH and stable pH of GST were determined as 30 °C, 100 mM using Tris-HCl, 7.80 using Tris-HCl and 5.50 using K-phosphate, respectively (Table 2).

**Figure 2:**
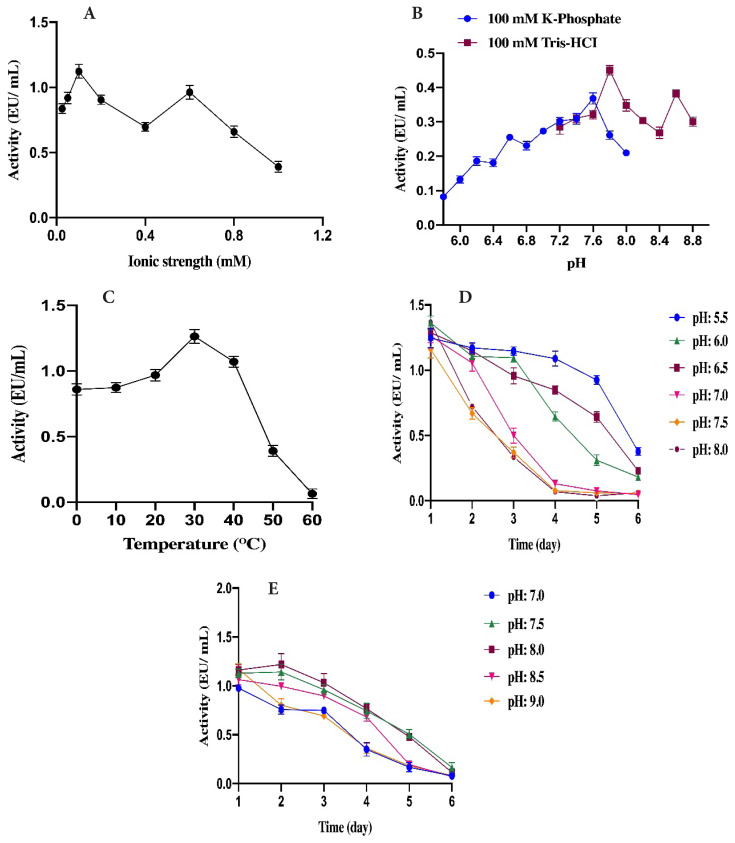
Characterization studies of *Chalcalburnus tarichi* Pallas liver GST. A-) Optimum ionic strength B-) Optimum pH C-) Optimum Temperature D-) Stable pH in K-phosphate buffers E-) Stable pH in Tris-HCI buffers.

There are similar results found in the literature. For instance, Çomaklı et al. [38] found the optimal pH, temperature, ionic strength, and stable pH for GST from rainbow trout erythrocytes as 10 mM K-phosphate, 7.30 using 0.01M K-phosphate, 7.30 using 0.01M K-phosphate, and 30 °C, respectively (Figure 2).

**Figure 3:**
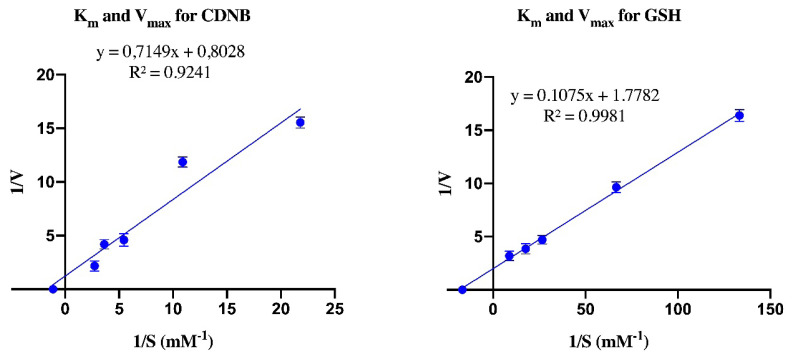
Kinetic analysis of *Chalcalburnus tarichi* Pallas liver GST activity.

The kinetic parameters, V_max_ and K_m_ were evaluated using GSH and CDNB as cosubstrates. Respectively, for GSH and CDNB, the V_max_ values were found as 0.562 and 1.245 EU/mL, and the K_m_ values were found as 0.06 and 0.89 mM (Table 2, Figure 3).

It was shown that GSH had a better affinity as a substrate in comparison to CDNB. Our results were like those obtained in bovine erythrocytes [39].

Recently, agricultural activities, rapid population growth, and industrial development have caused fish to be exposed to metal pollution [40,41]. Consequently, when heavy metals reach high concentrations in fish tissues, they become toxic. As the concentrations of metal ions in fish tissues increase, they bring about diminishing habitats of fish species. Consumption of fish with excessive heavy metal ions in its metabolism may give rise to various diseases such as diabetes, Parkinson’s, cancer, and Alzheimer’s. Understanding the metabolism enzyme inhibition is crucial for all living things. Most chemicals such as heavy metals and almost all drugs exhibit an interaction mechanism with an enzyme [42].

In this study, the inhibitory effects of Ag^+^, Cd^2+^, Co^2+^, and Cu^2+^ ions on GST enzyme activity were investigated. K_i_ constants and IC_50_ values were determined using plots. The IC_50_ values of Cd^2+^, Cu^2+^, Co^2+^, and Ag^+^ against GST were calculated as 0.235, 0.163, 0.446, and 0.00021 mM, and the K_i_ constants of these metal ions were determined as 0.117 ± 0.031, 0.049 ± 0.009, 0.893 ± 0.3, and 0.002 ± 0.0007 mM, respectively (Table 3).

**Figure 4:**
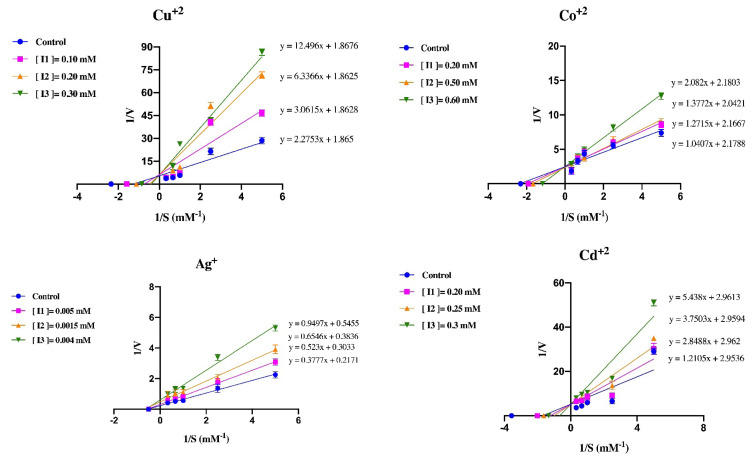
Lineweaver–Burk graph of Cd^2+^, Co^2+^, Cu^2+^, and Ag^+^ using three different metal concentrations for determination of K_i_ and inhibition type.

The inhibition mechanism of Ag^+^ was noncompetitive, while the inhibition mechanisms of Cd^2+^, Cu^2+^, and Co^2+^ were competitive (Figure 4). Cd^2+^, Cu^2+^, and Co^2+^ ions acted by binding to the active site of the GST enzyme and therefore showed competitive inhibition. On the other hand, Ag^+^ ions showed their effect by binding to a region other than the active site of the GST enzyme and therefore showed noncompetitive inhibition.

There are a few studies about interactions between GST and metal ions in the literature. For instance, in our previous study, GST was purified from Lake Van fish gills, and the effects of Cd^2+^, Cu^2+^, Zn^2+^, and Ag^+^ metal ions at different concentrations on in vitro GST activity were studied. We calculated K_i_ constants in the range of 0.433–1670 μM. Among the studied metal ions, Ag^+^ exhibited the best inhibitory effect [19]. In another study, Aksoy et al. [15] purified GST from muscle tissue by glutathione agarose affinity chromatography. They investigated the inhibitory effects of Cd^2+^, Pb^2+^, Ag^+^, Cu^2+^ Cr^2+^, Co^2+^, Fe^3+^, Zn^2+^, and Ni^2+^ metal ions on enzyme activity. They showed that Ag^+^ was the best inhibitor among all studied metal ions. GST was purified from turkey livers, and the researchers determined the inhibitory effects of Ag^+^, Mg^2+^, Cu^2+^, Hg^2+^, Fe^2+^, Zn^2+^, Mn^2+^, and Ni^2+^ on enzyme activity. They found that Ag^+^ exhibited a potent inhibitory effect [12]. These results were like ours. Additionally, Güller et al. [43] studied the effects of Ag^+^, Cu^2+^, Co^2+^, Cd^2+^, Zn^2+^ Pb^2+,^ Cr^2+^, and Fe^3+^ ions on GST enzyme activity in the liver of Bonito (*Sarda Sarda*). They reported that Ag^+^, Cu^2+,^ Cd^2+^, and Zn^2+^ showed inhibitory effects, but Cr^2+^, Fe^3+^, and Pb^2+^ did not show any impact on enzyme activity. The inhibitory effect order was determined as Cu^2+^>Ag^+^>Cd^2+^>Zn^2+^. The enzyme was inhibited noncompetitively by these metals.

Consequently, in this study, we purified GST from Lake Van fish liver and studied various characteristics such as the optimum pH, temperature, ionic strength, stable pH, V_max_, and K_m_. The specific activity of the enzyme was found higher than those determined in previous studies. The inhibitory effects of various heavy metals on GST enzyme activity were also studied. Findings in the relevant literature and our findings have showed that living organisms have varying levels of susceptibility to metal ions. These metal ions, which give rise to inhibition in GST enzyme activity on micromolar levels, may cause toxic effects in fish.

## Figures and Tables

**Table 1:** Purification steps of the GST.

Purification steps	Total volume (mL)	Activity (EU/mL)	Protein (mg/mL)	Total protein (mg)	Total activity (EU)	Specific activity (EU/mg)	Purification fold	Yield (%)
Homogenate	12.1	3.708	40	484	44.866	0.092	1	100
Glutathione-Agarose affinity chromotography	4.5	3.725	0.127	0.572	16.762	29.304	316.11	37.36

**Table 2:** Summary of characterization studies of *Chalcalburnus tarichii* Pallas liver GST.

Type of operation	Result
Optimum pH	7.80 (Tris/HCl)
Optimum ionic strength	100 mM (Tris/HCl)
Optimum temperature	30 °C
Stable pH	5.50 (KH_2_PO_4_)
Natural molecular weight	62.35 kDa
Subunit moleculer weight	33.80 kDa and 28.00 kDa
GSH K_m_	0.06 mM
GSH V_max_	0.56 EU/mL
CDNB K_m_	0.89 mM
CDNB V_max_	1.25 EU/mL

**Table 3:** IC_50_, K_i_ values and inhibition types for GST used GSH substrate.

Metal ions	IC_50_ (mM)	K_i_ (mM)	Inhibition type
Cd^2+^	0.235	0.117 ± 0.031	Competitive
Cu^2+^	0.163	0.049 ± 0.009	Competitive
Co^2+^	0.446	0.893 ± 0.3	Competitive
Ag^+^	0.00021	0.002 ± 0.0007	Noncompetitive
